# Role of p53 in the Regulation of Cellular Senescence

**DOI:** 10.3390/biom10030420

**Published:** 2020-03-08

**Authors:** Mahmut Mijit, Valentina Caracciolo, Antonio Melillo, Fernanda Amicarelli, Antonio Giordano

**Affiliations:** 1 Sbarro Institute for Cancer Research and Molecular Medicine, Center of Biotechnology, College of Science and Technology, Temple University, Philadelphia, PA 19122, USA; 2 Department of Medical Biotechnologies, University of Siena, 67100 Siena, Italy; 3 Department of Life, Health and Environmental Sciences, University of L’Aquila, 53100 L’Aquila, Italy

**Keywords:** p53, senescence, cell cycle arrest, microenvironment, DNA damage

## Abstract

The p53 transcription factor plays a critical role in cellular responses to stress. Its activation in response to DNA damage leads to cell growth arrest, allowing for DNA repair, or directs cellular senescence or apoptosis, thereby maintaining genome integrity. Senescence is a permanent cell-cycle arrest that has a crucial role in aging, and it also represents a robust physiological antitumor response, which counteracts oncogenic insults. In addition, senescent cells can also negatively impact the surrounding tissue microenvironment and the neighboring cells by secreting pro-inflammatory cytokines, ultimately triggering tissue dysfunction and/or unfavorable outcomes. This review focuses on the characteristics of senescence and on the recent advances in the contribution of p53 to cellular senescence. Moreover, we also discuss the p53-mediated regulation of several pathophysiological microenvironments that could be associated with senescence and its development.

## 1. Introduction

The concept of cellular senescence was initially introduced several decades ago by Leonard Hayflick [1]. He observed a phenomenon of “replicative senescence” in cultures of non-immortalized human fibroblasts and described it as the exhaustion and/or the termination of proliferative capacity of primary cells in culture [2]. Replicative senescence has been linked to progressive telomere shortening, a physiological function occurring every time the cell divides and which has been associated with the activation of specific molecular pathways [3,4].

Currently, senescence is considered an irreversible process of growth arrest occurring in response to cellular aging as well as to various sources of cellular stimulations, including activated oncogenes, cytokines, reactive oxygen species, DNA damage, or nucleotide depletion [5,6,7]. This form of senescence is also known as stress-induced premature senescence (SIPS) and has broad implications in health and diseases [8,9].

Recently, increasing interest has focused on the role of p53 in the regulation of cellular growth induced by intense oncogenic signals or replicative stress [10]. Upon stimulation, p53 regulates the expression of a large number of target genes involved in cell cycle arrest, DNA repair, senescence, and apoptosis [11]. Numerous studies show that p53 plays a critical role in the maintenance of genomic integrity through its role in DNA damage response [12]. Loss of p53 function promotes (directly and indirectly) chromosomal instability, inducing cells to enter either senescence or apoptosis [13].

A deep understanding of the mechanisms by which p53 is implicated in regulating cellular senescence is of great interest for the development of new therapeutic strategies. In this review, we provide an overview of the most recent discoveries in the field of cellular senescence. In particular, we focus on the p53-dependent senescence signaling pathways involved with different stages of senescence and with a consideration for the associated key biomarkers. We also provide an overview on the regulation of p53-mediated cellular senescence in the context of different pathophysiological conditions.

## 2. Senescence as Cellular Response to Stress

Cellular senescence is defined as a cell state characterized by prolonged and generally irreversible cell-cycle arrest [14] and the acquisition of different phenotypic alterations, including morphological changes, chromatin remodeling, metabolic reprogramming, and secretion of pro-inflammatory factors or SASP (senescence associated secretory phenotypes). These latter count cytokines, growth factors, proteases, and non-soluble extracellular matrix proteins [15]. All these features ultimately limit the replication of both old and damaged cells. Upon physiological conditions, proliferating cells commit to a regular cell cycle [15,16]. On the other hand, both physiological aging, characterized by telomere shortening, and long-term chronic stress, which impairs genomic integrity and stability, could lead to the activation of the senescence pathway [17]. In this sense, the senescence can be viewed as an adaptative response of cells and organisms when exposed to certain unfavorable environmental conditions.

Stressors include both intrinsic factors, such as oxidative damage, telomere attrition, hyperproliferation, oncogene activation, and environmental sources, including UV-light, γ-irradiation, and chemotherapeutic drugs [18,19]. Regardless of their origin, the stress factors trigger DNA damage responses (DDR) in the affected cells, which can result in different outcomes, depending on the cell type and the extent of the damage [20]. Mild DNA damage normally induces cell cycle arrest, while severe injury can activate the senescence program or the death programs; the latter includes apoptosis, mitotic catastrophe, autophagy, and necrosis [21].

p53 plays a pivotal role in determining the fate of the cells, and, in the context of senescence, its activation can take place in a DDR-dependent or DDR-independent way [22,23]. In the first case, telomere erosion, DNA damage, as well as hyperactivation of oncogenes and inactivation of onco-suppressors (oncogene induced senescence, OIS) resulting from replicative stress activate the DNA damage repair cascade [24]. DDR activates the stress sensors’ telangiectasia-muted (ATM) or ataxia telangiectasia and Rad3-related (ATR) kinases. ATM/ATR, in turn, activate the p53/p21^cip1^ axis by phosphorylating both p53 and its ubiquitin ligase Mdm2, leading to the stabilization of p53 levels [25]. P53 is directly phosphorylated in Ser-15 and indirectly phosphorylated in Ser-20 via Chk1/2 [26].

The importance of the DDR activation as an essential and causal factor in the activation of p53 and induction of senescence has been recently questioned. Many recent studies actually demonstrated how many OIS pathways can actually activate p53 bypassing the DDR: Ras through NOREA1, which promotes the pro-senescence acetylation of p53 while inhibiting its pro-apoptotic phosphorylation, AKT through the downregulation of MnSOD, onco-suppressor PTEN depletion, inducing mTORC1 and mTORC2 to bind to p53 instead of MDM2, and MAPK p38γ through direct phosphorylation of p53 [27,28]. These studies and the mechanisms they describe therefore show once again the crucial role of p53 and of p53-triggered senescence for the suppression of tumorigenesis after the onset of a first mutation.

## 3. Critical Cellular Senescence Pathways

**Figure 1:**
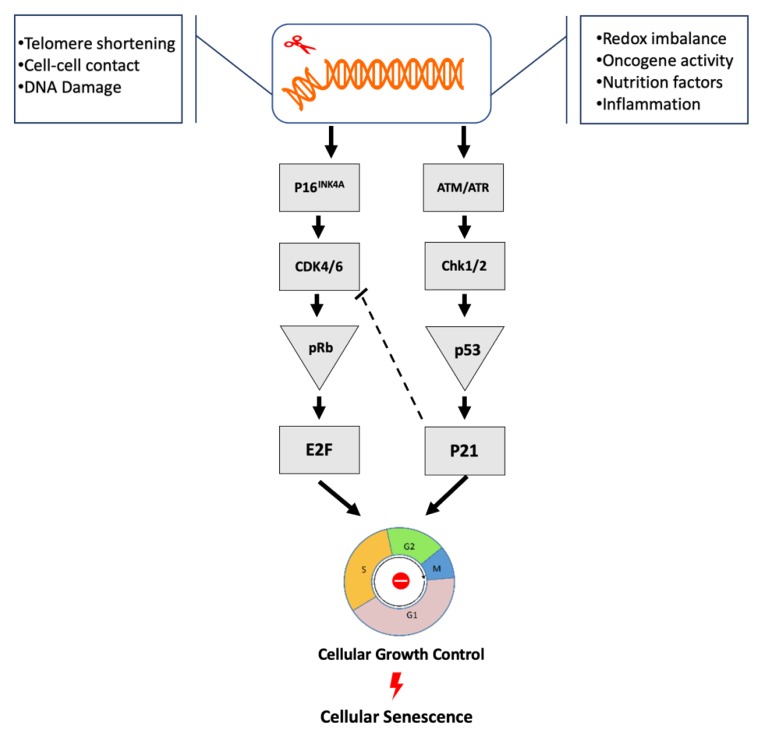
Induction and initiation of cellular growth control and senescence via p53 or P16^INK4A^ pathways. Various internal or external stress factors trigger the DNA-damage response (DDR) pathway, which in turn activates the p53 and/or the p16^INK4A^ pathways. p16^INK4A^ inactivates Cdk4/6, results in accumulation of phosphorylated pRb, stops the regulation of E2F transcription factors, and drives cell cycle arrest or senescence. These stressors also trigger DNA damage (cellular responses to such damages are regulated by either ATM-Chk2 or ATR-Chk1 pathways) and transactivate p53 and p21^CIP1^. Moreover, p21^CIP1^ protein levels may lead to the inhibition of Cdk4/6 activity, which contribute to the G1 arrest or senescence [33,34].

Crosstalk among cellular pathways is extremely important for the regulation of tissues and organ homeostasis, and this requirement appears to be maintained in senescent cells. The most extensively studied pathways involved in the regulation of cellular senescence are p53/p21^cip1^ and/or p16^INK4A^/Rb tumor suppressor pathways [29,30] (Figure 1). DNA damage and /or DDR seem to critically control the two pathways [31,32]. Therefore, identification (specificity) of senescence signaling pathways (pattern) in terms of senescent cause factors as well as in the microenvironment seem crucial for the initiation of senescence.

### 3.1. p53/p21^cip1^

p53 mainly functions as a transcription factor, upregulating or downregulating the expression of specific target genes by binding their promoter region, promoting alternative transcription by binding intronic sites, or interacting with coactivators such as the TATA box binding protein. A number of downstream effectors implement the p53-dependent modulation of the expression of these and other target genes. Fisher et al. tried to take a census of these genes and proposed approximately 3661 potential p53 targets [11]. This impressive number is a reflection of the many—sometimes alternative—roles p53 can play; in fact, several of these genes are known to be involved in the regulation of metabolism, autophagy, DNA damage repair, cell cycle arrest, quiescence, senescence, and apoptosis [35]. Consequently, research efforts aimed to identify which factors cause p53 to induce the expression or the repression of specific genes and therefore to activate a precise cellular response to a stress stimulus. In the induction of senescence, a crucial factor seems to be represented by the severity and the duration of the stress stimulus; senescence seems to require a stable stimulus, while transient stimuli just induce a transient growth arrest, allowing the cell to attempt to repair the damage. More severe stimuli lead instead to apoptosis [36]. The role of these parameters seems to rely mainly, but not only, on the effect on p53 levels and kinetics. Concordantly with this view, there is evidence of how these different p53-mediated programs are associated with different p53 levels. Chen and Liu et al. treated human diploid fibroblasts with increasing doses of H_2_O_2_ and, in result, they identified sublethal doses of H_2_O_2_ able to induce senescent-like growth arrest, while higher doses induced apoptosis. They evidenced how, in induced apoptotic conditions, p53 levels were two times higher compared to p53 levels in growth arrest conditions [37]. The threshold of stressor intensity between senescence and apoptosis was also proven to differ from cell type to cell type, and many types of cells were proven to prefer one solution over the other [38]. Regardless of the intensity, some stimuli seem to always trigger senescence; for example, busulfan, an alkylating agent, was proven to selectively induce senescence instead of apoptosis in WI38 fibroblasts [39]. The concentration of p53 is not the only parameter determining its activity. Alternative splicing of p53 mRNA and post-transcriptional modifications of p53 protein play an important role as well. Δ40p53, Δ133p53α, and p53β are the p53 isoforms mostly involved in cellular senescence, especially in its earlier stages. The biological and the physiological functions of these isoforms were extensively treated in a recent review by Fujita K [40]. Briefly, they hypothesized a model for the regulation of cellular senescence where Δ133p53α, abundant in proliferating cells, counteracts p53 functions while p53β levels are low. In senescent cells, p53β is upregulated by SRSF3-mdiated splicing, and Δ133p53α is downregulated by STUB1 mediated autophagic degradation. Moreover, Δ40p53 seems to regulate cellular senescence and aging by acting in two different ways: by directly regulating the IGF-1 signaling pathway to modulate cell growth and survival factors and by regulating the transcriptional activity of full-length p53 on target gene through direct binding of p53 full-length [40].

Acetylation is one of the most studied post-translational modifications of p53. Knight et al. proved how acetylation of p53 on certain sites prevents the phosphorylation of some serines in the p53 NH2-terminal region only, allowing the activation of genes with high p53 affinity, such as CDKN1A, which encodes for p21^cip1^, a crucial player in the senescence pathway. Acetylation in other sites leads to p53 hyperphosphorylation and therefore interaction with genes with low p53 affinity, such as pro-apoptotic genes [41]. Another important factor that seems to play a role in inducing p53-mediated senescence seems to be represented by the surrounding microenvironment; for instance, Kuilman et al. demonstrated that, in inflammatory conditions, IL-6 activity is necessary for both induction and maintenance of senescence induced by BRAF overexpression in human dermal fibroblasts. They also reported co-localization of IL-8 and senescence markers in human colon adenomas [42]. All these factors (p53 concentration, post-translational modifications, and microenvironment) are responsible for inducing, through p53 activity, a precise pattern of genetic expression, which, under the aforementioned conditions, leads to senescence. This pattern then displays itself through the activity of several effectors, which play different roles in the p53 senescence cascade, not only determining the cell cycle arrest and contributing to the expression of the senescence phenotype but also through the inhibition of the alternatives to the senescence program such as quiescence and apoptosis.

Among these effectors, the most important role is played by p21^cip1^. p21^cip1^ is the founding member of the mammalian CDK inhibitor family and, as such, it is required for the p53-induced cell cycle arrest at either G1/S or G2/M checkpoints [43,44]. Once activated, p21^cip1^ performs many functions, including its role in mediating the gene expression modulation of many p53 targets such as CDC25C, CDC25B, and survivin, mainly through the E2F4 complex recruitment [45]. However, the crucial importance of p21^cip1^ relies on its capacity of promoting senescence through the inhibition of apoptosis. p21^cip1^ was proven to bind many apoptosis agents, including many caspases. p21^cip1^ knockout in senescent cells provokes programmed cell death through the caspase activation cascade [46]. This is consistent with the evidence of the inverse relationship between apoptosis and p21^cip1^ levels, and the molecular mechanism underlying this notion was finally elucidated by Zhang et al., who demonstrated how, in response to high levels of doxorubicin, p53 inhibits p21^cip1^ expression through DNMT3a in colorectal cancer cells [47]. Furthermore, when Martinez et al. treated prostate cancer cells with antisense p21^cip1^ adenovirus, this led to the development of apoptosis at lower doses [48]. The research around this particular mechanism can give us just an example of the promising possible therapeutic applications that can derive from a deeper knowledge of all the different mechanisms linking alternative cellular programs such as senescence and apoptosis, many of which revolve around p53 and its downstream effectors. p21^cip^ is also capable of inducing senescence independently from p53 activity. Alionat-Denis et al. reported that Chk2 was able to induce p-21^Cip1^ expression in p53-defective cell lines, contributing to Chk2-mediated senescence [49].

Another study reported that deletion of p53 in Atm ^−/−^ mice abrogates early senescence in mouse embryonic fibroblasts (MEFs), but a high percentage of these mice die prenatally or develop tumors earlier than either single knockout. Accordingly, deletion of p21^cip1^ in Atm^−/−^ mice also abrogates early senescence in MEFs. Atm^−/−^ p21 cip1^−/−^ mice, similarly to Atm^−/−^ mice with deleted p53, are not more susceptible to tumors and exhibit a delayed onset of lymphomas, presumably due to increased lymphoid cell apoptosis, suggesting that p21^cip1^ is a critical participant in p53-dependent senescence pathways [50].

### 3.2. p16^INK4a^/Rb

While the p53/p21^cip1^ pathway seems to play a key role in the initiation of senescence, the pathway involving p16 and the retinoblastoma family of proteins (Rb family) seems to have a central role in the maintenance of senescence [2,51]. This was suggested by the observation of a decrease in p53 levels after induction of senescence, while p16 levels stayed steadily high [52]. In addition, Beausejour et al. demonstrated how the downregulation of p53 in senescent cells has different effects depending on p16 activity; it succeeds in inducing replication and cell growth in cells with low levels of p16, while it does not in cells with high p16 activity [53]. According to these findings, the activation of p16 pathway would be responsible for drawing a line between two different phases of senescence: an early, reversible phase dominated by p53 activity and an irreversible phase induced by p16/Rb pathway [54].

The p16 pathway can involve different proteins belonging to the Rb family, namely pRb/p105, p107, and pRb/p130 [55]. The different roles of these homologous proteins, which seem to be overlapping to a certain point, are still to be completely clarified, and we previously showed how it may very well depend on the cell line. However, in the progression of senescence in human cells, a primary role seems to be held by Rb2/p130 through the cyclin A repression, with the classic Rb involved just in the early phases [56]. The activation of the p53 pathway and the consequent cell cycle arrest context seem to be responsible for this shift, inducing the decrease in the levels of p105 and p107 and the increase in the levels of p130 in its hyperphosphorylated form, thus the proposal of p130 as a more specific senescence marker [57]. However, the relationship between p130 and p53 seems to display itself even more upstream in the regulation of the induction of replicative senescence. Our group pointed out the role of p130 in telomere shortening, which then induces the p53 activation [56].

The p53/p21^cip^ and the p16^INK4a^/Rb pathways appear to be constantly interacting and influencing each other through many crosstalk mechanisms that are more or less elucidated. For example, induction of senescence can be prevented by inactivation of p53 prior to upregulation of p16. However, once p16 is highly expressed, downregulation of p53 cannot reverse cell cycle arrest [58]. In fact, the crosstalk between p53 and pRb demonstrated that, when p53 and pRb were restored concomitantly to normal levels in human cervix carcinoma cells, cellular senescence was induced in nearly all of the cells [59].

### 3.3. Senescence as a Multistep Process

The classical depiction of senescence as a static, uniform, and irreversible cellular state has been progressively reconsidered, and senescence is now envisioned as a dynamic and multistep process [60]. During the initiation of the senescence, which is also called as “primary senescence”, the stressed cells may be still able to repair and/or eliminate the cause of the damage and then can escape from cell cycle arrest. For example, Dirac and Bernards demonstrated that a small proportion of senescent embryonic fibroblasts were capable of reentering the cell cycle when p53 expression was suppressed through RNA interference. Time-lapse photomicrographs showed that the cells reentering the cell cycle were originated from senescent cells [61]. However, these rare cases are considered to take place only in the early stage of senescence establishment. Moreover, persistent exposure to a damaging environment leads the cells to the next stage of senescence, known as “developing senescence”, where cells are poised to demonstrate full-featured senescence [62]. Nevertheless, if senescent conditions continue for extended periods of time, as it happens, for instance, in the aging process, the cells enter a third phase of senescence, known as “late senescence”, in which they may be characterized by heterogeneous phenotypes [63]. Many studies described phenotypical and transcriptional heterogeneity of senescent cells in both in vitro and in vivo models. Additionally, while a significant contribution to this heterogeneity was shown to be dependent on the different types of cells, time-series transcriptomic profiling studies also showed the expression of different genes at different times [64]. Characteristic alterations of senescent cells include flattened and enlarged cell shape, expanded lysosomal compartment and vacuoles, increased metabolic rate and reactive oxygen species (ROS) production, senescence-associated secretory phenotype (SASP) secretion, nuclear and chromatin alterations, and resistance to apoptotic stimuli [65,66]. In most of the models studying senescent cells, p53 (as well as the DDR proteins) seems to be involved in the earlier stages, and the time factor plays an important role in the entire process [67]. p53 activity decreases with time, and this is consistent with the idea of p53 (and p21^cip1^) being a crucial factor for the induction of the senescence and a gate to an early phase and still reversible senescence [68]. On the other hand, the subsequent increase of p16 activity would be responsible, as suggested by Beauséjour et al., for a late senescent state characterized by a distinct and permanent senescence phenotype, which is no longer reversible through p53 inhibition [53]. The vision of the senescence as a dynamic process is very intriguing. To date, however, the experimental evidences are based on results and observations obtained from a very narrow set of cellular models, and more studies are needed to support this new concept.

A summary of the most common pathways and biomarkers associated with different phases of senescence is reported in Table 1.

## 4. Targeting the p53-Dependent Senescence in Different Pathophysiological Environment

Cellular senescence has been implicated not only in aging itself but also in the onset of age-related disorders, including atherosclerosis, diabetes, and Alzheimer’s disease, in cancer, and, more recently, in biological processes such as tissue repair and regeneration and homeostasis of the microenvironment [72,73]. Its role is not restricted to cell growth control but also affects cell-to-cell interactions. In fact, through the secretion of SASP, senescent cells can affect the surrounding cells in different microenvironments. In this context, tissue specific differences can influence different p53 expression, contributing to the determination of the cell fate after exposure to genotoxic stress [74].

Here, we further discuss the participation of p53-mediated cellular senescence in different pathophysiological conditions (Table 2).

### 4.1. Cancer

Cellular senescence is a physiological mechanism adopted by cells to limit tumorigenesis [99,100], and for this reason, a therapy-induced senescence was considered as an alternative and possibly safer approach to the traditional cancer therapy, which aimed at inducing extensive DNA damage [101]. In this regard, modulation of p53 activity was also taken into consideration. Cancer cells, unlike other cells, exhibit high frequency of mutations in the p53 gene. p53-dependent upregulation of p21^cip1^ leads to dysregulation of DNA replication and has been reported in aggressive cancer cells [101]. The p53/p21^cip1^ axis in the context of cellular senescence has been implicated in several cancer types, including breast cancer, cholangiocarcinoma, head and neck squamous cell cancer, liver cancer, lung cancer, colorectal cancer, as well as ovarian cancer [102]. It was reported that reactivation of p53 in tumors provokes tumor regression mediated by induction of senescence [103]. Moreover, small molecules were developed to increase the amount or the activity of p53 in cancer cells [104]. For example, Nutlin stabilizes p53 by inhibiting p53 interactions with MDM2 [105]. Another study reported that the inhibition of the interaction between p53 and MDM4, a negative regulator of p53, restores the activity of p53 in melanoma cells, resulting in an increased sensitivity to cytostatic or cytotoxic therapy [104]. The idea of reactivating p53 to reduce the tumor progression is very appealing, and many therapeutic strategies have been under study. However, their applications in clinics are still very difficult, mainly due to the complexity of the p53 regulatory network and the molecules involved. In most cases, therapies aimed at reactivation of p53 will be part of a combined therapy in which the biggest challenge will be finding the best therapeutic partner in relation to the specific tumor type and the molecular status of p53 [106].

The downside of this senescence-inducing approach is that senescent cells often align with malignant cells and support tumor expansion [104] as well as cancer initiation and progression [107]. Senescent cells regulate tumorigenesis and the immune response of their neighbors in both a positive and a negative manner via SASP and depending on the genetic context, thus limiting the application of this therapy [104].

### 4.2. Metabolic Disorders

The term “metabolic disorders” refers to different types of pathophysiological conditions involving disruption of metabolic homeostasis and are always associated with higher prevalence of age-related diseases, including cardiovascular diseases, obesity, and diabetes. Metabolic and signaling changes seen in diabetes, such as high circulating glucose, altered lipid metabolism, and growth hormone axis perturbations, can promote senescent cell formation [108]. There is evidence supporting that cellular senescence in the vascular system, named “vascular senescence”, is critically involved in the pathogenesis of cardiovascular and metabolic disorders [109].

p53 plays a vital role in the endothelium environment and contributes to the maintenance of endothelial homeostasis. However, senescent endothelial cells change their morphological and functional characteristics and cannot correctly regulate repairing and regenerative activities of endothelial progenitor cells (EPCs) [110]. The p53-p21^cip1^ pathway appears to be more responsive than the p16-Rb pathway for the induction of endothelial cell senescence. This is because the knockdown of p53 expression, but not p16, inhibits endothelial cell senescence induced by a variety of stimuli. Similar results were found in an in vitro study [111], where the blood flow-induced mechanical forces determined arterial injury and promoted endothelial senescence via p53-p21^cip1^-dependent pathways [112]. In addition, a recent study showed that oxidized small and dense diabetic low-density lipoprotein (LDL) accelerate the onset of senescence of EPC recovered from normal subjects via p53-mediated signaling pathways. This was further confirmed by another study, in which depletion of endogenous p53 in EPC recovered from diabetic patients prevented the accelerated onset of senescence, indicating that a p53-mediated pathway contributes to the impaired EPC function in this setting [113]. Overall, p53-mediated endothelium senescence results in endothelial microenvironment dysfunction and further disrupts the balance of endothelial barriers, which is not only associated with the onset of metabolic diseases themselves but also contributes to the development of its complication.

### 4.3. Inflammatory Responses and Inflammation-Associated Diseases

Inflammation is the immune system’s response to harmful stimuli, such as pathogens, damaged cells, toxic compounds, or irradiation, and represents a defense mechanism that aims to restore the body homeostasis. It is responsible for many chronic diseases, including cardiovascular and bowel diseases, diabetes, arthritis, and cancer [114,115]. The connection between inflammation and cellular senescence was initially suggested by studies on the gene expression profiles of senescent cells, where inflammatory responses were associated with wound healing processes [116]. In inflammatory conditions, the senescence mechanism can be activated by either oncogene or inflammatory mediators. Alternatively, the induction of inflammatory network is also linked to premature senescence induced by oncogenes. Increased expression of the inflammatory regulators has been observed in primary human diploid fibroblasts induced to undergo senescence by oncogenic *ras* (*H-ras^V12^*) [117].

Chronic obstructive pulmonary disease (COPD) is a chronic inflammatory disease characterized by an obstructed lung airflow affecting normal breathing. The causal factors might be attributed to smoking as well as other air pollutants. Indeed, many cellular senescence markers, including p53, p21^cip1^, and p16, were found in both the airway epithelium and the endothelium of subjects with COPD [118]. A study by Sundar and colleagues revealed that the murine model of cigarette smoke (CS) can induce chronic lung epithelium inflammation, and that further triggers cellular senescence via a p53-p21^cip1^ that does not require p16 [119].

Although cellular senescence itself is a cell-autonomous process, it has profound effects on neighboring cells/tissues via the action of SASP mediators. The SASP profile may be unique and may ultimately determine whether senescence serves useful purposes or contributes to disease pathology [118]. The important role of the SASP inflammatory response in tumor prevention was demonstrated in mouse models for hepatocellular carcinoma (HCC), where induction of senescence by p53 activation in malignant hepatocytes was shown to reduce tumor size by SASP-mediated recruitment of immune cells to the tumors [120].

What happens to the inflammatory senescence in the absence of p53? p53 has been demonstrated to inhibit inflammatory responses, and functional loss of p53 causes excessive inflammatory reactions [121]. For example, a significant number of p53-null mice die before tumor development from inflammation, resulting in abscesses, gastroenteritis, or myocarditis [122]. Senescence induced in oncogene-expressing cells is a p53-dependent tumor-suppressor mechanism that prevents malignant transformation by suppressing cellular proliferation [121]. In addition, senescence is also characterized by secretion of a set of cytokines and chemokines known as the senescence-associated secretory phenotype (SASP) by constitutively active NF-kB [123]. Therefore, p53 might function as a restrictor and attenuator of inflammatory reactions via the balance between p53 and NF-kB.

### 4.4. Neurodegenerative Diseases

Different studies have aimed at the identification of senescent cells in the brain and at the understanding of their role in the pathophysiology of neurodegenerative diseases. These age-related pathologies are characterized by great heterogeneity, and for this reason, a primary causal role of cellular senescence in these diseases seems unlikely. However, cellular senescence may still contribute to disease susceptibility, age at disease presentation, and rate of progression [118]. In a recently published study, Baker et al. demonstrated the presence of senescent microglial cells and astrocytes in their experimental mice of neurodegenerative disease and evidenced how such cells led to neurodegenerative diseases and memory problems [124]. Other reports have linked senescence to the development of aging-related neurodegenerative diseases in human patients [125]. Within these perspectives, the pharmacological elimination of senescent cells could represent a beneficial therapeutic approach for the treatment of these pathologies. It is then important to understand the mechanisms through which senescent cells affect the normal brain functioning.

Neuroinflammation is a common feature for the onset of several neurodegenerative disorders, and it is an important contributor to Alzheimer’s disease (AD) and Parkinson’s disease (PD) pathogenesis and progression. Neuroinflammation is always accompanied by an increase in SASP-expressing senescent cells of non-neuronal origin in the brain [126].

Astrocytes can exert toxic effects or protective effects on neurons. Neurotoxic effects of astrocytes are mediated by SASP involving pro-inflammatory cytokine secretion (e.g., Il-6), while neuroprotection is mediated by neurotropic growth factors such as NGF [126]. Turnquist et al. reported the expression of two isoforms of p53 in astrocytes, ∆133p53 and p53β; in in vitro primary human senescent astrocytes, a decreased expression of the isoform ∆133p53 was reported, and the decreased expression of this isoform, linked to neuroprotection, was attributed to autophagic degradation [127]. These findings suggest that regulatory mechanisms of p53 isoforms may represent a potential target for therapeutic strategies.

Increase in p53 level and activity was observed in PD patient brains as well as in PD animal and cellular models, and that mostly correlated with DNA damage, activated cellular stress response, and apoptosis. Bussian et al. reported an accumulation of p16^INK4A^ positive senescent astrocytes and microglia in a mouse model of tau-dependent neurodegenerative disease, and the clearance of senescent glial cells prevented tau-dependent pathology and cognitive decline [128]. Taken together, these results demonstrated that the disease progression was dependent on extracellular signaling from senescent microglial cells expressing p16^INK4A^, posing the premises to develop strategies to halt or revert neurodegenerative diseases.

## 5. Conclusion Remarks

In the past few decades, great efforts have been made to elucidate the mechanisms through which p53 regulates the cell fate. It is now abundantly clear that the cellular responses to p53 activation depend on multiple factors, including the nature of the cell stressors (e.g., DNA damaging radiation, chemicals, telomere erosion, oxidative stress, osmotic shock, deregulated oncogene expression, ribonucleotide depletion), the cell lineage, the cell physiological conditions, and the cellular environment. Aside from inducing cell growth arrest and apoptosis, activated p53 also modulates senescence, apparently displaying a dual effect of promoting or, in some cases, inhibiting the senescence program.

Cellular senescence disables the proliferative potential of damaged cells. Nevertheless, although senescent cells are considered to be permanently arrested, they are still metabolically active and secrete a variety of molecules with pro-inflammatory or proteolytic activity to communicate with the tissue microenvironment and the neighboring cells. This way, senescent cells can attract and interact with inflammatory cells and can be eliminated by the immune system. The presence as well as the clearance of senescent cells from the organism are beneficial to physiological and pathological processes such as tissue homeostasis and regeneration, wound healing, protection against cancer, or embryonic patterning, overall improving survival and recovery from aging related disorders. This aspect has offered the opportunity for therapeutic interventions aimed at increasing the elimination of senescent cells for the prevention of continuous tissue damage or to prevent senescent cells harboring oncogenic mutation from re-entering the cell cycle as cancer cells. Beside their protective activity, senescent cells can also exert detrimental effects, due both to their persistent accumulation in the tissues and to the nature of the biomolecules they secrete. They can contribute to the formation of a pro-tumor microenvironment and can induce chronic inflammation and organ degeneration. For this reason, more research is needed to define when and where senescent cells are beneficial or instead induce adverse consequences; in this sense, taking into account the diversity of a tissue specific microenvironment may elicit a distinctive nature of senescence. Hence, a major challenge is to increase the number and the reliability of markers to identify senescent cells in the context of different microenvironments. Understanding the underlying mechanisms involved in interactions with normal tissue and in the removal of senescent cells in each particular microenvironment will be critical to better exploit different and more effective therapeutic strategies.

## Figures and Tables

**Table 1:** Cellular senescence dynamic development.

Senescence Phase	Description of the Involved Pathways	Associated Markers	Reference
Primary senescence	Induction of p53/p21 pathway, induction of antiproliferative transcriptional program	BAF57, GADD45 NOTCH1/N1ICD, p21	[62,69]
Developing senescence	p53/p21 and/or p16 pathway, SASP release, Morphological changes	p21, P19, p16, LIMA1, Ki-67	[70,71]
Late senescence	Overproduction of SASP, chromatin remodeling, CCFs formation lysosomal activity, p16 pathway	Il-6, PGC-1β, SA-beta-galactosidase IFN-I, Ki-67	[14,60]

**Abbreviation**: BAF57: subunit of the BAF (BRG1-Associated Factor) complexes; GADD45: growth arrest and DNA damage; NOTCH1/N1ICD: notch intracellular domains (N1ICD and N2ICD); CCFs: cytosolic chromatin fragments; PGC-1β: peroxisome proliferator-activated receptor; IFN-I: interferon (IFN)1; SASP: senescence-associated secretory phenotype.

**Table 2:** The mechanism summary of p53-mediated cellular senescence in different diseases.

Diseases Category	Diseases	p53 Regulation in Disease	Induction of Senescence	Pathological Characterization	Reference
Cardiovascular metabolic disorders	Cardiovascular injury	+	PAI-1; MEOX2	caveolin-1 Inhibits VSMCs grow and promote senescence; Meox protein play a role in p53-mediated endothelial disfunction	[75,76]
	Obesity	−/+	HFD	Body weight phenotype and behavioral disorders; p53 represses the lipogenic Srebf1 pathway in Adipocytes	[77,78]
	Diabetes	+	hyperglycemia	p53 contributes to insulin resistance; decreased islet proliferation	[79,80]
Neurodegenerative diseases	Parkinson’s disease	+	MPTP; αSyn fibrils	The KOp53 mice in Parkinson’s disease model	[81,82]
	Alzheimer’s disease (AD)	+	Aβ (1-42)	SIRT3 rescues neurons from p53 mediated senescence; Aβ (1-42) induces senescence	[83,84]
	Huntington’s disease	+	CAG144 R6/2	P53, miR-34a was disrupted in R6/2 mouse brain tissue; senescence involved in the striatum during HD development	[85,86]
Cancer/ tumors	Breast cancer	+	RSV; Kindlin-2	RSV metabolites induce senescence in breast cancer cells	[87,88]
	Skin Tumor	+	N-WASP; Doxycycline	N-WASP is a negative regulator of senescence induction by p53	[89,90]
	Brain cancer	+	TGFβ	Acute activation of senescence in Glioblastomas	[91,92]
Inflammation	Musculoskeletal pain	+	Glucocorticoids; Hsp90β	Induction of irreversible senescence; Hsp90β/MDM2 induced p53-dependent senescence on muscle regeneration	[93,94]
	Asthma/bronchitis	+	LPS; TNF-α	cell senescence promotes chronic lung inflammation; downregulation of ITGB4 induces senescence in inflammation	[95,96]
	COPD	+	lncRNA1	accelerated cellular senescence in COPD	[97,98]

**Abbreviation**: PAI-1: plasminogen activator inhibitor-1; Meox2: mesenchyme homeobox 2; HFD: High Fat Diet; Srebf1: Sterol Regulatory Element-Binding transcription factor1; MPTP: 1-methyl-4-phenyl-1,2,3,6tetrahydropyridine; CAG144 R6/2: transgenic mice model human Huntington’s disease (HD); RSV: resveratrol; WASp: the Wiskott-Aldrich syndrome protein; TGFβ: transforming growth factor beta;; LPS: lipopolysaccharides; TNF-α: tumor necrosis factor; ITGB4: integrin β4; COPD: chronic obstructive pulmonary disease.
